# Components and Biological Activities of Venom from Lionfishes (Scorpaenidae: *Pterois*)

**DOI:** 10.3390/md23020055

**Published:** 2025-01-23

**Authors:** Candelario Rodriguez, Jafeth Carrasco, Gaspar Bruner-Montero, Osmindo Rodrigues Pires Júnior, Marcelino Gutiérrez, Edgardo Díaz-Ferguson

**Affiliations:** 1 Estación Científica Coiba-AIP, Ciudad del Saber, Clayton, Panama 0816-02852, Panama; crodriguez@coiba.org.pa (C.R.); gbruner@coiba.org.pa (G.B.-M.); osmindo@unb.br (O.R.P.J.); 2 Centro de Biodiversidad y Descubrimiento de Drogas, Instituto de Investigaciones Científicas y Servicios de Alta Tecnología-AIP, (INDICASAT), Ciudad del Saber, Clayton, Panama 0843-01103, Panama; 3 Sistema Nacional de Investigación, Secretaría Nacional de Ciencia, Tecnología e Innovación, Ciudad del Saber, Clayton, Panama 0816-02852, Panama; 4 Centro de Biología Celular y Molecular de Enfermedades, INDICASAT, Ciudad del Saber, Clayton, Panama 0843-01103, Panama; jcarrasco@indicasat.org.pa; 5 Laboratory of Toxinology, Department of Physiological Sciences, Institute of Biological Sciences, University of Brasilia, Brasilia 70910-900, DF, Brazil

**Keywords:** lionfish, marine toxins, venomous fish, *Pterois*, Scorpaenidae

## Abstract

Fishes of the genus *Pterois* possess spines that provoke intense pain, which can last for weeks. Since the first toxicological description of their spine venom, a significant amount of research has been published regarding their biochemical characterization. This minireview presents research published from 1959 to 2024 on bioactive substances found in *Pterois* species. *Pterois* venom mainly contains peptides and proteins that display a range of biological activities, including anticancer, antimicrobial, antioxidant, antiviral, enzymatic, cardiovascular, procoagulant, neurological, neuromuscular, and nutraceutical effects. Although *Pterois* venom contains bioactive substances, the toxic side effects, such as hemolysis and nociception, of these venoms should be considered. Hence, further intense research is needed to establish the potential uses of *Pterois* venom for human health.

## 1. Introduction

**Figure 1:**
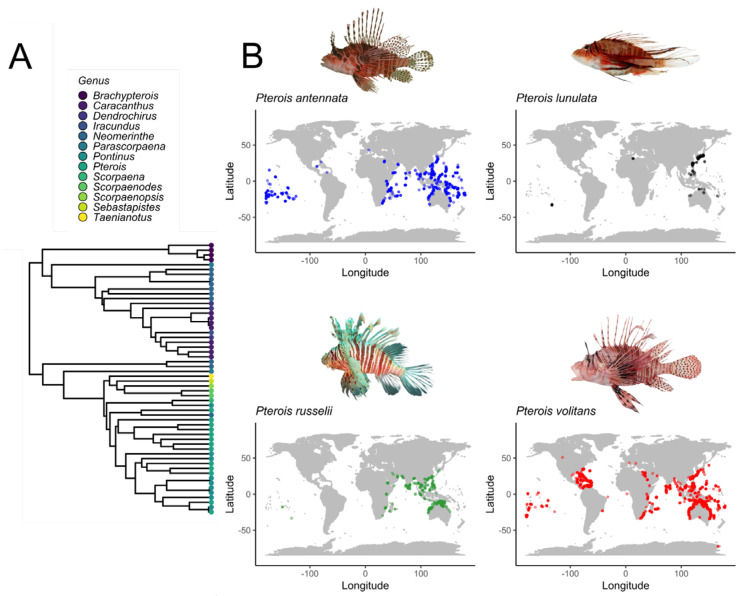
Phylogeny of the family Scorpaenidae. The phylogenetic tree represents the evolutionary relationships among genera within the family Scorpaenidae. The tree was constructed using fossil-calibrated data from the Fish Tree of Life (fishtreeoflife.org) [12]. The data were retrieved and handled using the R programming language (version 4.3.0) and visualized with the ggtree package. Tip points are color-coded to represent different genera; the corresponding genera are displayed in the legend (**A**). Geographical distribution of some *Pterois* species (**B**). Occurrence data were taken from FishBase.org (accessed 28 September 2024). Copyright permissions were obtained from Nok-Wai Lai and Lorraine Brennan.

Nature can be envisioned as a drugstore, with living organisms, such as plants, endophytic microbiota, and venomous organisms, being sources of templates for drug development. Since ancient times, animal toxins have been included in the medicinal practices applied by tribes worldwide [1]. Fish venoms have evolved through various types of natural selection, such as predation, competition, and microbial pathogens, which may have promoted their biological activity and chemical diversity [2]. Remarkably, natural products obtained from venomous marine and terrestrial animals have biomedical applications, and some animal toxins are under preclinical evaluation and in advanced clinical phases [3]. Indeed, animal toxins have provided molecules that are being employed as FDA-approved drugs for treating different diseases such as cancer and microbial infections [1]. According to phylogenetic analysis, many venomous marine organisms are fishes since approximately three thousand species of fish are considered venomous [4]. However, there has been much less attention given to the development of drugs from venomous fish sources than from microorganisms, marine invertebrates, and organisms of plant origin [5]. Toxins can be used by the animal that produces them as a passive or active defense mechanism [6]. *Pterois* fishes deliver toxins to victims through wounds caused by the spines [7]. *Pterois* fishes are found mainly in the Indo-Pacific Ocean; however, in recent decades, invasions have been noted, and these species can now be found in most oceans (Figure 1). Among these invasion events, the one in the Caribbean, Gulf of Mexico, and North Atlantic coast of the United States was initially managed as a species complex *P. volitans*/*P. miles* invasion and later as a single species, given the predominant presence of *Pterois volitans* (Linnaeus, 1758); there have been several invasions reported, with genetic evidence for these events [8,9,10,11]. 

**Figure 2:**
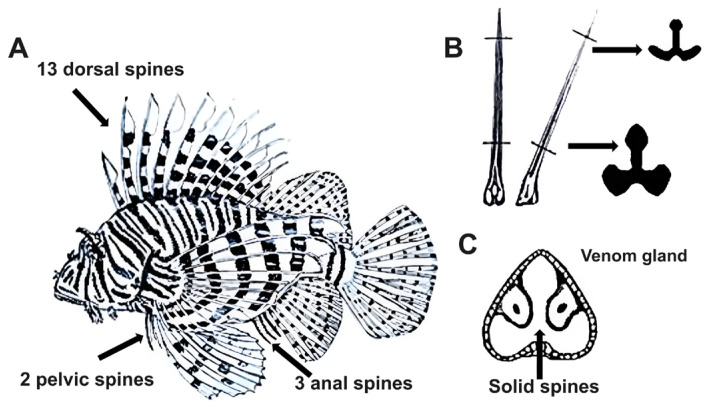
Venomous apparatus of *Pterois* species: (**A**) Adult *Pterois* specimen and its venom spines. (**B**) Spine structure. (**C**) A cavity on the spine that contains venom.

In the fish of the genus Pterois, the venom apparatus consists of spines, which are associated with an epidermis layer containing secretory cells. Such cells produce toxins in their fins as an active defense mechanism, with the main purpose of protecting the fish from predators. There are eighteen venomous spines (thirteen dorsal, three anal, and two pelvic), each of which is thin and enveloped in integumentary sheaths. These spines are often retractable and contain two longitudinal grooves connected to venom-secreting tissue, which enables toxin transport and release [13]. Lionfish spines are solid and composed of a dentine-like substance, and they possess a trilobed morphology; the cross-section of a dorsal spine resembles an inverted T-shaped solid structure (Figure 2). This trilobed shape is formed by two anterolateral grooves along the upper two-thirds of the spine’s length, which house glandular tissue covered by a thin membrane that ruptures upon penetration to release the venom [14]. Pterois venom-secreting tissue comprises a complex arrangement of secretory cells specialized for venom production and storage. Microscopic examination of cross-sections of the venom gland revealed a cluster of large polygonal glandular cells with a pinkish-gray, fine granular cytoplasm [15]. Chemically, the venom includes various bioactive substances, such as neurotoxic peptides, proteolytic enzymes, and lipid compounds, which contribute to its potency and lethality [14].

Toxicological evaluations of *Pterois* venom have revealed its clear pharmacological effects such as hemolysis and pain. Envenomation by lionfish occurs mainly in the hands of people upon cleaning the home aquarium or during net fishing recovery. Among the features observed after *Pterois* punctures are (in order of frequency) marked pain and local edema, paresthesia, abdominal cramps, and extensive edema. Additionally, fewer than 50% of victims develop other symptoms, such as tachycardia, skin rash, gastrointestinal disorders, weakness, and hypertension, and approximately 20% develop local infections caused by bacteria [16]. Biochemical analysis of Balb/c mice intravenously injected with crude *Pterois* spine venom at sublethal doses (LD_50_: 210 µg/mouse) revealed increases in the levels of the organ damage markers alkaline phosphatase, alanine transaminase, creatine kinase, lactate dehydrogenase, and serum aspartate transaminase. The levels of all enzymes increased during the first 3 h, reaching a maximum at 24 h, which was followed by a rapid decrease after 48 h. Moreover, histological studies of these organs indicated that *Pterois* venom induces cardiotoxic, hepatotoxic, nephrotoxic, and pneumotoxic effects [17]. In fishes of the *Synanceia* genus, the toxicity of spine venom has been attributed to a protein with a molecular weight of 150 kDa; however, the toxic protein from *Pterois* is still unknown [18]. The lethality and hemolysis of lionfish spine venoms from *Pterois antennata* (Bloch, 1787), *Pterois lunulata* (Temminck & Schlegel, 1843), and *P. volitans* were neutralized by stonefish *Synanceia trachynis* (Linnaeus, 1766) antivenom [19]. Lionfish envenomation is treated by immersing the affected area in warm water (45 °C) for one hour; however, this method is not always effective [20]. Complications, such as bacterial infections and those that are neurological in nature, are frequently relieved with pharmacologic intervention, including antibiotics and painkillers [21]. In biomedicine drug discovery, determining the therapeutical window of bioactive substances before further analysis, such as in vivo assays and clinical studies, is crucial. Peptides and proteins are biomolecules that act through cytoplasmic membrane targets and can potentially be the next generation of first-line drugs against different diseases. However, this fact can be limited by some factors, such as digestive lysis caused by proteases, potential inactivation by cations, carbohydrates, and mucin, and the scant knowledge of the mechanism of action, in addition to the limited understanding of the structural activity relationship behind the in vitro bioactivity observed. In the case of bioactive proteinaceous substances from *Pterois*, no research progress has been reported [22].

In this manuscript, we review the reported data concerning the chemical characterization and pharmacological evaluation of crude, fractionated, and purified substances from species of the genus *Pterois*. Additionally, future directions are proposed regarding the use of these peptides and proteins in drug discovery.

## 2. Crude Preparation, Fractionation, and Chemical Components from *Pterois* Venom

Active extracts of Pterois spines can be prepared by extracting the spines with a buffer solution or distilled water [23]. Nevertheless, the extraction of natural mixtures such as lionfish venom is challenging because of the presence of enzymes such as lipases and proteases that may degrade the cooccurring bioactive substances. Toxins from marine fish are mainly proteinaceous substances. Chemical modifications of the secondary structures, such as the α-helices and β-sheets, of peptides and proteins can result in misfolding and hence the loss of bioactivity [24]. Furthermore, the structure of the Pterois spine makes the extraction and recovery of venom a nontrivial task, making the preparation of the initial crude material a crucial step. However, several methods have been tested to accomplish this. The dorsal spine extract obtained with 40% glycerol in water and stored at −85 °C was stable for one year and retained 90% of its bioactivity [25]. Compared with extraction with 0.5% glycerol, the use of protease inhibitors helps increase the stability of Pterois venom extracts, as demonstrated by the retention of bioactivity and a greater protein recovery (0.84%). The recovery percentage did not increase when glycerol was mixed with protease inhibitors. This methodology should be optimized since protease inhibitors may further interfere with biological tests, such as enzymatic assays [26,27]. On the other hand, it is crucial to remove the glycerol from the crude extracts before the active components are purified; in this sense, size exclusion chromatography or dialysis results in the efficient recovery of proteins [28]. The extraction of Pterois venom with organic solvents (such as n-butanol) has also allowed the isolation of low-molecular-weight bioactive substances [29]. Since proteins from venomous marine fishes, including Pterois, are often unstable, some strategies have been developed to increase the stability of the proteins by decreasing the entropy of the peptide chain. One such method consists of replacing other amino acids with proline residues, which results in β-turn structures [30]. Another method involves introducing disulfide bonds and, hence, maintaining the native structure of the protein [31].

After the initial extraction, fractionation of the crude extract by partition or other fractionation procedures is recommended. Size exclusion chromatography (diol column) via high-performance liquid chromatography (HPLC) allowed the starting material to be separated into four main fractions with up to 13% protein recovery. Reversed-phase HPLC (silica bound to C4 chains) was applied, and several active fractions were obtained. Among the bioactivities reported to have been retained are protease, hyaluronidase, and anticancer activities [27]. Saturation by salting out has proven to be an efficient method for concentrating bioactive components from Pterois venom. Extraction with 80% ammonium sulfate afforded proteins with anticancer and antimicrobial properties and 40 to 60% antioxidant activity [32,33,34].

### 2.1. Peptides

**Figure 3:**
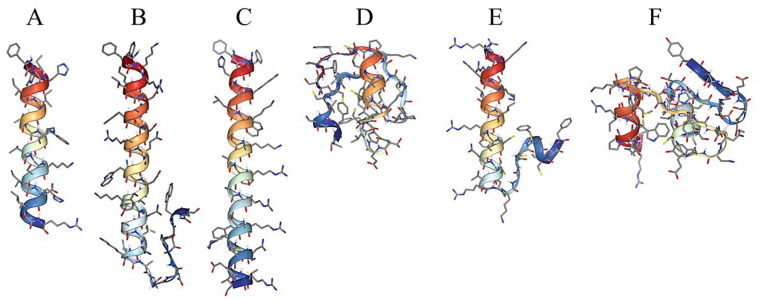
Predicted structures of the small molecules and peptides found in *Pterois* species: (**A**) Pteroicidin-α. (**B**) Pteroicidin-β. (**C**) Pteroicidin-γ. (**D**) PV-defensin. (**E**) PV-hepcidin. (**F**) PV-leap2. The secondary structures of the peptides were modeled by employing the software PEP-FOLD (version 4) with visualization considering the pH and ionic strength in an aqueous solution.

The venom extracted from the spines of *Pterois* contains molecules with low molecular masses (<1000 Da), also termed secondary metabolites, because of their ecological roles, such as in defense and reproduction. In lionfishes, these molecules may be important, although their roles are not well understood. The venom from *P. volitans* contains an ichthyotoxin with a molecular weight of 327 Da and an unknown structure. This toxin has only been identified in living fish [29]. Additionally, acetylcholine and an unknown neurotoxin were identified in the aqueous fraction of the spines from *P. volitans* [23]. Additionally, ten small molecules were detected in the spine venom of *P. volitans* via mass spectrometry. However, structural elucidation based on mass spectrometry was not supported by any database or additional strategies for chemical characterization; hence, the identification should be considered tentative [35,36]. Peptides have been detected in *P. volitans*. β-Defensin, a cationic peptide with 42 amino acid residues and a molecular mass of 4495.39 Da, was identified in its pelvic spines. This peptide was also detected in the transcriptome of venom from spines along with five other cysteine-rich peptides: pteroicidin-β, pteroicidin-γ, hepcidin (molecular mass of 3001.58 Da), LEAP-2 (46 amino acid residues) and NK lysin (153 amino acid residues) [37]. The skin of *P. volitans* also contains two bioactive peptides, pteroicidin-α(COOH) and pteroicidin-α(CONH_2_), with molecular weights of 2409.09 Da and 2408.22 Da, respectively [38]. The biological activities of proteinaceous substances are influenced by their net charges and secondary structures. Cationic peptides can interact with components of microbial membranes. In addition, the α-helical structure has been shown to be involved in antimicrobial effects through the formation of pores or micellization [39]. Some regions of antimicrobial peptides found on *Pterois* venom adopt mainly α-helical conformations (Figure 3).

### 2.2. Enzymes

Most of the proteins in lionfish venom have molecular weights of less than 100 kDa. However, the proteins in the venom from *Pterois russelii* (Bennett, 1831) and *P. volitans* have molecular masses ranging from 6 to 200 kDa [27,40]. *Pterois* venom proteins have been identified in partially purified bioactive fractions (Table 1). Biochemical characterization of the proteins purified from *Pterois* venom has resulted in the identification of their enzymatic activities. Crude venoms from the dorsal spines of *P. antennata* and *P. volitans* have hyaluronidase activities. In general, *P. antennata* contains lower amounts of peptides (38–98 units/mL) than *P. volitans* (217–421 units/mL). In both cases, the hyaluronidases displayed optimal activity in 0.1 M NaCl at 37 °C and pH 6.6 and were specific for the substrate hyaluronic acid. The identities of both hyaluronidases are quite similar, each having 483 amino acid residues and only two differences at positions 102 (Val/Asp) and 429 (Ser/Arg) [41]. Venoms from *P. volitans* were collected separately, and it was found that dorsal spines showed high hyaluronidase and proteolytic activity, whereas the caudal spines lacked hyaluronidase activity but displayed strong proteolytic activity against gelatin. According to zymographic analysis, hyaluronidase from *P. volitans* has an apparent molecular weight between 55 and 72 kDa, and some bands with proteolytic activity were observed in this range. Furthermore, proteases between 34 and 43 kDa and approximately 19 kDa in size were detected via zymography using gelatin [42]. A protease with a molecular weight of 45 kDa was detected in the venom of *P. volitans* [43]. The effects of pH and temperature on the protease activity of *P. volitans* venom were analyzed, and the proteases were active under neutral and basic conditions. On the other hand, heating to 60 °C denatures the enzymes [44]. Proteolytic activity was identified in venom from *P. russelii* in a 96-well microplate containing casein. The activity was dependent on the dose and reached a maximum absorbance with 1.88 µg of crude venom [40]. Some authors have reported that *Pterois* venoms do not have phospholipase activity [42,43]. However, the venom from *P. russelii* was shown to have phospholipase A2 activity starting at 10.32 µg, and its maximum activity was observed at 80 µg [40]. Furthermore, phospholipase A2, with a molecular weight of 85 kDa, was detected in the venom of *P. volitans*. The enzyme-specific activity was determined to be 13.872 units/µg [45]. Although semi-purified fractions containing bioactive proteins can be obtained upon fractionation employing gel electrophoresis and salting out strategies, optimizing the chromatographic conditions is encouraged. High-performance chromatographic techniques such as size exclusion, reversed-phase, and ionic chromatography serve as efficient methodologies for animal venom toxin research [46]. These experimental results show that *Pterois* fish venom is a source of enzymes that can be isolated. However, deeper investigations should focus on the bioprospection, kinetics, stability, and structure-activity relationships of these substances, as specificity and efficiency are valuable features that only enzymes offer and can be exploited in relevant fields such as biomedicine [47]. In this sense, some challenges, such as contamination by the presence of other components of the venom and the low amounts of protein produced, which limit the potential of these enzymes in biotechnology, can be addressed by heterologous enzyme expression in bacteria [48].

## 3. Biological Activities of *Pterois* Venom

This section describes the reported biological activities of proteins identified in the venom of *Pterois* fishes. Mechanisms of action are described for some substances; however, more research is needed in this field. Furthermore, the reported components of *Pterois* venom have been identified mainly from the dorsal spines, but shorter venomous spines, such as the pelvic and anal spines, have been found to contain bioactive substances [42].

### 3.1. Antimicrobial Activity

Although the antifungal and antiprotozoal activities of *Pterois* venom are still unknown, the activities of these venoms against bacteria have been reported. The antibiotic potential of the venom of *P. volitans* against human pathogens has been described. Crude venom did not inhibit *Staphylococcus aureus*; however, fractionation with 80% ammonium sulfate at 100 µg/mL resulted in an inhibition halo of 14.35 mm according to the disk diffusion method employing chloramphenicol at 30 µg/disk as the positive control (inhibition halo of 26.46 mm) [33]. The fraction obtained with 80% ammonium sulfate was then heated at 60 °C for 35 min and tested against *Escherichia coli* and *Salmonella* sp. via the agar dilution method. The evaluation of this fraction at 3.77 µg/mL revealed 98.81% and 89.8% inhibition, respectively. Chloramphenicol at 1000 ppm (complete inhibition) was used as a positive control [49]. In both studies, the antibiotic fractions exhibited phospholipase A2 activity. Phospholipases can form pores and increase the permeability of the bacterial membrane [33,49]. Antimicrobial peptide sequences have been identified in RNA from *Pterois* venom. Pteroicidins belong to the piscidin family, which are peptides found in fish skin that are associated with host antimicrobial defense. Pteroicidin-α, pteroicidin-β and pteroicidin-γ have been identified in *P. volitans*. Pteroicidin-α was shown to have antibiotic activity, with MIC values between 5 and 50 µM against *S. aureus*, *E. coli*, and the fish pathogen *Aeromonas salmonicida*. The antibiotic mechanism of pteroicidins was investigated, but no positive control for inhibition was reported. Pteroicidin-α-CONH_2_ displays bactericidal activity, whereas pteroicidin-α-COOH (nonamidated) is a bacteriostatic compound. Additionally, the C-terminal amide group of pteroicidin-α increases its toxicity, as seen in hemolytic assays [38]. Lower hemolytic activity was found among shorter fragments of pteroicidin-β and pteroicidin-γ. The antimicrobial evaluation revealed that fragments containing 17 to 20 amino acid residues display broad antibiotic activity, whereas smaller fragments (13–10 amino acid residues) inhibit E. coli only [37].

Molecular docking between proteins from *P. volitans* and *Helicobacter pylori* revealed chemical interactions. For the analysis, hyaluronidase and FV proteins from *P. volitans* were employed, and they were tested against the virulence proteins from *H. pylori* Cag A, Cag L, Cag D, GGT, and urease. In silico analyses revealed the strongest interaction between *P. volitans*’ hyaluronidase and Cag A from *H. pylori*. Among the interactions between the two proteins, five salt bridges, ten Pi interactions, and thirty-six hydrogen bonds were observed at atomic distances that range from 2.6 to 5.4 Å [50]. The peptides from *Pterois* venom revealed promising antibiotic activity in vitro; however, a cytotoxicity evaluation should be conducted to investigate their true potential. In contrast to the common antibiotics employed in clinical trials, which act on intracellular targets, antimicrobial peptides have received much attention since they inhibit the cell membrane of bacterial pathogens [51].

### 3.2. Anticancer Activity

Marine organisms are great potential sources for anticancer drug development. Some anticancer compounds from marine sources are already on the market and in clinical trials [52]. Few studies have been carried out with venomous fishes of the Scorpaenidae family as sources of anticancer candidates [53]. Venom obtained from the spine of *P. volitans* has been shown to have inhibitory effects on several cancer cell lines in both in vitro and in vivo models. The effects of different doses of the venom crude extract were evaluated in Swiss Webster strain mice bearing Ehrlich’s ascites carcinoma xenografts. After treatment with crude venom at a dose of 4.25 µg/kg, the lifespan of the mice increased by 99.5%, and the tumor volume decreased, displaying 0.8 mL in comparison with those of the negative control group (4.5 mL) [54]. Venom from *P. volitans* extracted with 60 and 80% ammonium sulfate was evaluated and showed 19 and 17% inhibition, respectively, against HeLa cervical cancer cells at 2.5 mg/mL [55]. The anticancer activity of the 80% ammonium sulfate fraction was improved by heating at 70 °C for 30 min as the bioactivity of the fraction at a dose of 0.75 mg/mL increased to 37.79% [32]. Remarkably, the protein FV purified from *P. volitans* venom was shown to have anticancer effects against HeLa and alveolar epithelial cancer cells in vitro. Treatment with 2 µg/mL FV for 24 h selectively inhibited the growth of both cancer cell lines, and no toxic effects to normal human lymphocytes were observed. The mechanism of action of FV involves apoptosis, as indicated by the expression of caspase-8 and caspase-3, as well as the downregulation of the Bcl-2 protein [56]. Stonustoxin isolated from the venomous fish *Synanceia horrida* (Linnaeus, 1766) inhibited the development of the breast cancer cell line MCF-7, with an IC_50_ of 8.27 µg/mL [57]. The therapeutic window of the FV peptide and the apoptotic mechanism by which this protein acts addressed the first steps in its drug development. The next steps should include the evaluation of animal models. These findings highlight the underexplored potential of the Scorpaenidae family, particularly *Pterois* venom, as promising therapeutics for cancer treatment.

### 3.3. Antioxidant Activity

Radical oxygen and free radical species are produced during metabolism and can cause biomolecules to decompose. Oxidative stress is closely related to different pathologies, such as cancer, stroke, and neurodegenerative diseases. Several antioxidant substances have been approved for clinical use to treat several ailments. Natural antioxidants include small molecules such as uric acid, ascorbic acid, and melatonin, as well as enzymes such as catalase and superoxide dismutase [58]. Protein extracts prepared from the venom of *P. volitans* have been shown to have antioxidant properties using the 2,2-di-phenyl-1-picrylhydrazyl (DPPH) method. The extract obtained with 80% ammonium sulfate was tested at 2 mg/mL and displayed 57.08% inhibition. The antioxidant activity, calculated as the 50% inhibitory concentration (IC_50_), was determined to be 1.56 mg/mL. Purification of the crude extract with ethanol produced a similar protein content of 309.83 µg/mL; however, the extract showed very low antioxidant activity [36]. A combination of precipitation and heating at 75 °C revealed that the fraction obtained with 40–60% ammonium sulfate displayed 76.13% inhibition at 2 mg/mL, and the IC_50_ was calculated to be 1.31 mg/mL [34]. Although these values represent weak antioxidant activity (less than 200 µg/mL), further fractionation of the bioactive components could be optimized by employing different methods other than alcohol precipitation to increase purity [59].

### 3.4. Antiviral Activity

Viral diseases affect people worldwide and can cause millions of deaths when medications and vaccines are not available, as observed for every pandemic that has occurred. Although viruses can be described biologically, complex infection mechanisms, high replication rates, and their ability to mutate present great challenges. Antiviral proteins are a promising domain since these proteins can interact with viral proteins and host cells to promote viral inhibition in different ways [60]. Proteins and crude extracts of the venom from *P. volitans* displayed antiviral activity against simian retrovirus 2 (SRV-2). At 4 ppm, the crude venom inhibited viral replication by 84.20% in A549 cells infected with SRV-2. Extraction of the venom with 80% ammonium sulfate resulted in the purification of phospholipase A2 (PLA2), which has antiviral activity and displayed 97.78% inhibition at 4 ppm. Both samples exhibited dose-dependent antiviral effects. Furthermore, the crude venom did not present any cytotoxic effects at the bioactive concentration, and PLA2 showed only 21% inhibition of epithelial A549 human cells in the MTT assay [61]. Antiviral activity was also detected after the venom was extracted with caprylic acid and 20% ammonium sulfate. SDS-PAGE revealed that this fraction contained a protein with a molecular weight of 85 kDa and phospholipase activity; its antiviral activity reached 98.13%, and the lethal concentration against A549 cells was 28.36 ppm [45]. In comparison, antiviral phospholipases have been found in the venoms of snakes from different genera, such as *Crotalus*, *Bothrops*, *Oxyuranus*, and *Naja*. These secretory phospholipase A2 proteins have molecular weights of approximately 14 kDa, and their virucidal mechanism of action involves interference with host cell components [62].

### 3.5. Cardiovascular Activity

Cardiovascular diseases are the leading cause of death worldwide, of which several manifestations and risk factors are known. Among them, atherothrombosis is closely related to myocardial infarction, peripheral vascular disorders, and stroke [63]. The spine venom of *P. volitans* has shown bioactivity in the cardiac tissues of animal models. Isolated buffalo sculpin fish hearts were treated with *P. volitans* venom, and at low concentrations (40 µg protein/mL), a positive chronotropic effect was observed by an increase in the heart rate, whereas at higher concentrations (100–200 µg protein/mL), a hypotensive effect was produced, and at 1 mg of protein/mL, an irreversible standstill occurred [26]. Administration of *P. volitans* venom at a medium dose (130 µg of protein/kg) caused a decrease in blood pressure and did not affect the heart rate or electrocardiogram of Webster Swiss albino mice in vivo. At a higher dose (200 µg of protein/kg) of *P. volitans* venom, bundle branch block, extrasystole, ventricular fibrillation, and ventricular tachycardia were observed [25]. At a concentration of 0.618 µg protein/mL, *P. volitans* venom caused relaxation of the porcine coronary arteries in vitro. Additionally, a dose of 10 µg/mL produced a decrease followed by an increase in the contractile force of the rat atria. The effects occur in part via muscarinic and β1-adrenergic receptors [64]. The evaluation of purified toxins from other Scorpaenidae fishes, such as verrucotoxin (*Synanceia verrucosa* Bloch & Schneider, 1801), stonustoxin (*Synanceia horrida*), and Sc-Tx (*Scorpaena plumieri* Bloch, 1789), revealed hypotensive effects in animal models at doses of 16, 20 and 70 for each toxin, respectively [65]. The potential cardiovascular activities of understudied *Pterois* species remain to be explored. Some proteins with cardioactive activity have been identified in the venom of *P. volitans*; however, more in-depth studies of their identity, toxicity, and potential uses in pharmacology are suggested (Table 1).

### 3.6. Coagulant Activity

Venoms from some marine fishes display procoagulant properties. Fish venom from the stingray *Himantura imbricata* promotes coagulation in vitro measured by the microtiter plate method, with a prothrombin time (PT) of 16 s and a partial thromboplastin time (PTT) of 158 s at a dose of 2 µg (final volume 150 µL) [66]. *Scorpaena* and *Synanceia* fishes are not known to have coagulant activities; however, venoms obtained from the spines of *Pterois* fishes have been reported to possess procoagulant properties [67]. The crude venom extract of *P. russelii* displayed coagulant activity in human plasma (obtained from a healthy donor) in a dose-dependent manner. At 30 µg, the clotted blood displayed a PT and PTT of 7 s and 14 s, respectively, with coagulant activities reaching 50% for PT and 44% for PTT. Final volumes were 300 µL and 600 µL for PT and PTT, respectively [40]. *P. volitans* venom showed coagulant activity against the blood serotypes O, B, A, and AB, with PT values of 15.5, 16.0, 14.5, and 16.0 s, respectively. Additionally, analysis of type A with different doses of the 80% ammonium sulfate protein fraction revealed a PT of 13 s and a PTT of 21 s at a dose of 7.5 µg (final volume 320 µL). The known procoagulant agent L-N-omega-nitroarginine methyl ester was tentatively identified in the crude venom [35].

### 3.7. Neurological and Neuromuscular Activity

Venomous animals produce toxins with an affinity for the nervous system that can be analyzed to detect molecules with potential neurological applications. Neurological substances can act by promoting or inhibiting chemical signals in synapses [68]. *Pterois* venom and purified fractions have shown neurological activity in animal models and human cells. In zebrafish embryos, treatment with *P. volitans* venom at 200 µg/mL induced the expression of acetylcholinesterase mRNA and increased the level of the α2 subunit of the nicotinic acetylcholine receptor. Remarkably, 1 µg of a fraction (F2) obtained from HPLC purification of the venom reversibly blocked the human neuronal α3β2 nicotinic acetylcholine receptor by 56.99 ± 5.50%. On the other hand, this fraction showed no activity against the human α7 acetylcholine receptor, suggesting that *P. volitans* venom displays differential bioactivity among neuronal receptor subunits [69]. The use of nicotinic acetylcholine receptors as drug targets has been suggested because of their implications in several diseases. For example, α3β2 nicotinic acetylcholine receptors are related to pathologies in the habenula, cerebellum, spinal cord, retina, and autonomic ganglia [70]. Substances that can protect the nervous system or reverse damage caused to neuron cells, termed neuroprotective agents, have been proposed for the treatment of neurodegenerative pathologies such as Alzheimer’s, Parkinson’s, and Huntington’s diseases [71]. The crude venom of *P. volitans* (administered orally at 42.5 µg/kg-w) displayed neuroprotective effects in rats after alcohol exposure. It was determined that the venom decreases oxidative stress and maintains a high level of acetylcholine, as observed in brain tissue [72]. Although prospective studies on neurological activity have focused on *P. volitans*, it has been suggested that other *Pterois* species may have components with neurological effects.

Toxins from Scorpaenidae venom display neuromuscular activity. Some of these substances have been found to cause neuromuscular blockage, which manifests as muscle relaxation, and have a promising future in anesthesia. Stonustoxin purified from *S. horrida* and evaluated in rat skeletal muscle blocked the nerves and muscles at doses between 8 and 50 µg/mL [73]. In comparison, the crude venom from *P. volitans* (30 µg protein/mL) caused pronounced contraction of the chick biventer cervicis muscle. The neuromuscular effect behind this response was investigated by measuring the intracellular molar concentrations of Ca^2+^ ions in mouse neurons. After treatment with venom (100 µg protein/mL), an increase in intracellular Ca^2+^ of up to 300% was observed in comparison with the basal values. This effect was potentiated after the addition of Ca^2+^ channel blockers but inhibited by the removal of extracellular Ca^2+^ or replacement with La^3+^. This observation suggests that the intracellular concentration of Ca^2+^ may be affected by the formation of pores in the cell membrane [74]. The venom from *P. volitans* causes muscle fibrillation in *Rana pipiens* nerves and skeletal muscle. This effect was due to the presence of micromolar concentrations of acetylcholine. However, after the venom was treated with acetylcholine esterase, muscle fibrillation was maintained, and neuromuscular transmission was subsequently blocked, suggesting that the bioactivity of the venom was due to an unidentified toxin. The membrane permeability was thus investigated; however, according to an artificial lipid bilayer assay, *P. volitans* venom is not able to form ion channels to increase membrane permeability [23]. Few neuromuscular drugs from natural sources have been developed despite the production of substances with neuromuscular activity being produced by venomous animals such as fish. Despite the demonstrated neuromuscular properties of *Pterois* venom, some barriers to its application need to be overcome, such as its myotoxic activity and the fact that most neuroprotective agents that have reached clinical phases are small molecules [71]. In this sense, identifying the specific region responsible for the neuromuscular activity of a bioactive protein may increase its therapeutic potential. Another strategy that has resulted in promising results in animal models involves modifying specific sites of the molecular framework to alter the physical properties, such as isoelectric point, and hence decrease toxicity [75]. Moreover, the incorporation of poly(ethylene glycol) groups has reduced the toxicity of several bioactive proteins [76].

### 3.8. Nutraceutical Activity

Nutraceutical therapy is the medical application of foods or supplements that may contain molecules such as peptides and proteins that, after being hydrolyzed by digestive enzymes, display biological activities against diseases [77]. Two monomers (α and β) of the previously identified 75 kDa toxin in the venom of *P. volitans* were investigated for nutraceutical applications, using a bioinformatics approach [78,79]. After in silico digestive hydrolysis, both subunits produced peptides with medicinal properties. The peptides VP, PL, AF, AF, AH, and DN showed antidiabetic activity by inhibiting the diabetes-related enzymes DPP-III and DPP-IV. The tripeptide VPL exhibited antihypertensive activity, as indicated by its ability to promote the release of vasoactive substances. The peptides SDF, AH, and EL showed antioxidant activity, and the dipeptides SF and TF were shown to inhibit renin. BLAST alignment revealed that these proteins are also present in the venoms of *P. antennata* and *P. lunulata* [79].

**Table 1:** Bioactivity of proteins identified in the venom of *Pterois* spp.

MM (kDa)	Identification ^d^	Lionfish	Biological Activity ^e^	Reference
7.6	MS	*P. volitans*	anticancer	[56]
7.9	PAGE	*P. volitans*	anticancer, antioxidant	[34]
14.0	PAGE	*P. volitans*	anticholinergic	[69]
14.2	SEC	*P. volitans*	*	[80]
15.7	PAGE	*P. volitans*	anticholinergic	[69]
19.0	PAGE	*P. volitans*	protease	[42]
26.0	PAGE	*P. volitans*	protease	[27]
29.0	PAGE	*P. volitans*	anticancer, cardioactive	[26]
32.0	SE	*P. volitans*	hemorrhagic	[80]
33.0	PAGE	*P. volitans*	anticancer	[56]
35.0	PAGE	*P. volitans*	*	[69]
39.2	PAGE	*P. volitans*	anticholinergic	[69]
45.0	PAGE	*P. volitans*	cardioactive, protease	[26,27]
46.2	PAGE	*P. volitans*	antioxidant	[34]
49.0	PAGE	*P. volitans*	anticancer	[56]
52.7	PAGE	*P. volitans*	antioxidant	[34]
53.3 ^a^	DNA Cloning	*P. antennata, P. volitans*	hyaluronidase	[41]
60.0	PAGE	*P. volitans*	protease	[27]
65.0	PAGE	*P. volitans*	anticancer	[56]
66.0	PAGE	*P. volitans*	cardioactive	[26]
73.0	PAGE	*P. volitans*	protease	[27]
75.0 ^b^	PAGE	*P. volitans*	hemolytic, hyaluronidase	[27,69,78]
80.0 ^c^	PAGE	*P. volitans, P. lunnulata*	protease	[27]
85.0	PAGE	*P. volitans*	antibiotic, anticancer, antiviral, phospholipase	[32,33,56,61]
90.6	PAGE	*P. volitans*	*	[69]
97.0	PAGE	*P. volitans*	cardioactive	[26,69]
100.0	PAGE	*P. volitans*	protease	[27]
110.0	PAGE	*P. volitans*	anticancer	[32]
111.0	PAGE	*P. volitans*	protease	[27]
116.0	PAGE	*P. volitans*	cardioactive	[26]
153.5 ^a^	DNA cloning	*P. antennata, P. volitans*	*	[78]
160.0	SE	*P. lunnulata*	hemolytic	[81]
200.0	PAGE	*P. volitans*	protease	[27]

^a^ Apparent molecular mass (MM) for 483 and 1397 aa residues [1 amino acid (aa) residue~110 Da]. ^b^ Monomer of the 153.5 kDa protein. ^c^ Monomer of the 160 kDa protein. ^d^ Technique used to characterize molecular weight: mass spectrometry; PAGE, polyacrylamide gel electrophoresis; or SE, size exclusion chromatography. ^e^ In vitro activity or activities of the fraction in which the toxins were identified. * Not reported.

## 4. Conclusions and Future Directions

Research on venom from *Pterois* fishes, although not abundant, highlights the biotechnological potential of these venoms, especially due to the presence of substances with biological activities in different areas of interest for human health. An analysis of the components of *Pterois* venom revealed antibiotic, anticancer, antiviral, and neuromuscular bioactivities. Further research should include an evaluation of the bioactive components for cytotoxicity to determine selectivity, which would warrant in vivo experiments. Moreover, purification of the active ingredient components in areas where *Pterois* venom has shown effects, such as cardiovascular, antioxidant, and coagulant activities, remains underexplored.

## 5. Recommendations

There have been ecological concerns regarding the uncontrolled invasion of lionfish, especially red lionfish (*Pterois volitans*), which have caused several negative impacts on Caribbean coral reef ecosystems and have the potential to colonize other important biodiversity areas worldwide, including the Eastern Tropical Pacific. However, the venom of these animals remains a promising source for the discovery of new drugs and biomedicines. In this context, the removal of these animals for bioprospection and biomedical purposes could minimize or even reverse the negative cascade of effects caused by their invasion, thus providing additional reasons for their control.

## Data Availability

No new data were created or analyzed in this study. Data sharing is not applicable to this review.
